# Supplementary material for: Ovule Transcriptome Analysis Discloses Deregulation of Genes and Pathways in Sexual and Apomictic Limonium Species (Plumbaginaceae)
Source: Genes (Basel). 2023 Apr 12;14(4):901. doi: 10.3390/genes14040901 (PMC10137852; doi:10.3390/genes14040901)
Supplement: Supplementary file 1 [file genes-14-00901-s001.zip › supplementary table S2.pdf]

Table S2. Uniquely annotated top down- and up-regulated differentially expressed genes (DEGs) in ovules from apomictic *Limonium multiflorum* (M) in stage S1 (1), relative to the control *L. auriculifolium* (a) ovules in either S1 (1) or S2 (2) stages, and *L. multiflorum* (M) in S2 (2) relative to *L. auriculifolium* (a) in S3/S4 (4). DEGs were annotated according to the *Arabidopsis thaliana* reference genome and are presented in ascending order of $\log_2$ fold-change ($\log_2$FC).

Gene ID	Gene Name	Protein Name	Molecular Function	$\log_2$FC
		**M1a1**		
		**Down-regulated**		
TRINITY_DN5225_c0_g1	*DIR1*	Putative lipid-transfer protein DIR1	lipid binding; protein binding; ion binding	-9.80
TRINITY_DN40200_c0_g1	*AT5G33370*	GDSL esterase/lipase At5g33370	hydrolase activity	-8.88
TRINITY_DN253_c1_g1	*COBL10*	COBRA-like protein 10		-8.86
TRINITY_DN7779_c0_g1	*AP3*	Floral homeotic protein APETALA 3	transcription regulator activity; protein binding; DNA binding	-8.66
TRINITY_DN8286_c0_g1	*DRT100*	DNA damage-repair/toleration protein DRT100	nucleotide binding	-8.62
TRINITY_DN8621_c0_g1	*AT59*	Probable pectate lyase 3	metal ion binding; lyase activity	-8.60
TRINITY_DN1267_c0_g1	*PEX4*	Pollen-specific leucine-rich repeat extensin-like protein 4	ATP binding; transferase activity	-8.50
TRINITY_DN4212_c0_g1	*NOI4*	Protein NOI4		-8.47
TRINITY_DN7954_c0_g1	*PI*	Floral homeotic protein PISTILLATA	transcription regulator activity; protein binding; DNA binding	-8.44
TRINITY_DN185471_c0_g1	*AT1G30350*	Probable pectate lyase 4	metal ion binding; lyase activity	-8.37
		**Up-regulated**		
TRINITY_DN44719_c0_g1	*IQM5*	IQ domain-containing protein IQM5		7.37
TRINITY_DN31216_c0_g1	*RL6*	Protein RADIALIS-like 6	transcription regulator activity	7.52
TRINITY_DN36508_c0_g1	*WRKY53*	Probable WRKY transcription factor 53	transcription regulator activity; DNA binding	7.62

TRINITY_DN41214_c0_g1	*ATM*	Serine/threonine-protein kinase ATM	ATP binding; DNA binding; transferase activity	7.84
TRINITY_DN38061_c0_g1	*ZFP8*	Zinc finger protein 8	transcription regulator activity; ion binding; DNA binding	8.04
TRINITY_DN35799_c0_g1	*MSL4*	Mechanosensitive ion channel protein 4	transporter activity	8.44
TRINITY_DN13495_c0_g1	*AT2G23090*	Uncharacterized protein At2g23090		9.13
TRINITY_DN28989_c0_g1	*WAKL13*	Putative wall-associated receptor kinase-like 13	ATP binding; carbohydrate binding; transferase activity	9.50
TRINITY_DN3514_c2_g1	*AT1G50180*	Putative disease resistance protein At1g50180	ADP binding; ATP binding	9.69
TRINITY_DN44488_c0_g1	*PRB1*	Pathogenesis-related protein 1		10.21
**M1a2**				
**Down-regulated**				
TRINITY_DN40200_c0_g1	*AT5G33370*	GDSL esterase/lipase At5g33370	hydrolase activity	-7.61
TRINITY_DN4445_c0_g1	*CEP1*	KDEL-tailed cysteine endopeptidase CEP1	hydrolase activity	-7.42
TRINITY_DN19789_c0_g1	*AT3G01270*	Probable pectate lyase 7		-7.32
TRINITY_DN9597_c0_g2	*MSBP1*	Membrane steroid-binding protein 1	lipid binding	-7.30
TRINITY_DN9027_c0_g3	*AP1*	Floral homeotic protein APETALA 1	DNA binding; transcription regulator activity; protein binding	-7.24
TRINITY_DN22841_c0_g1	*PXG4*	Probable peroxygenase 4	ion binding; oxidoreductase activity	-7.14
TRINITY_DN5730_c0_g1	*PME38*	Putative pectinesterase/pectinesterase inhibitor 38		-7.12
TRINITY_DN9027_c0_g1	*SEP1*	Developmental protein SEPALLATA 1		-7.09
TRINITY_DN3934_c0_g1	*AT3G07850*	Exopolygalacturonase clone GBGA483		-6.94
TRINITY_DN8621_c0_g1	*AT59*	Probable pectate lyase 3		-6.91
**Up-regulated**				

TRINITY_DN32666_c0_g1	*ARI8*	Probable E3 ubiquitin-protein ligase ARI8		8.40
TRINITY_DN17343_c0_g1	*MYB63*	Transcription factor MYB63		8.44
TRINITY_DN42433_c0_g1	*CRK32*	Putative cysteine-rich receptor-like protein kinase 32		8.78
TRINITY_DN7799_c0_g1	*AT1G06650*	1-aminocyclopropane-1-carboxylate oxidase homolog 3		8.93
TRINITY_DN13495_c0_g1	*AT2G23090*	Uncharacterized protein At2g23090	metal cluster binding; oxidoreductase activity; FAD binding; ion binding	9.12
TRINITY_DN6834_c1_g1	*DREB1D*	Dehydration-responsive element-binding protein 1D	DNA binding; transcription regulator activity; protein binding	9.31
TRINITY_DN40977_c0_g1	*ATG8C*	Autophagy-related protein 8c		9.52
TRINITY_DN4104_c0_g2	*LAC3*	Laccase-3	ion binding; oxidoreductase activity	9.93
TRINITY_DN44488_c0_g1	*PRB1*	Pathogenesis-related protein 1	hydrolase activity	10.21
TRINITY_DN68_c0_g1	*PRMT14*	Probable histone-arginine methyltransferase 1.4	transferase activity; protein binding	10.27
		**M2a4**		
		**Down-regulated**		
TRINITY_DN1473_c0_g1	PRE6	Transcription factor PRE6	isomerase activity	-8.30
TRINITY_DN11282_c0_g2	IAA7	Auxin-responsive protein IAA7	DNA binding; transcription regulator activity	-7.13
TRINITY_DN3927_c0_g1	AT3G28060	WAT1-related protein At3g28060	transporter activity	-6.62
TRINITY_DN8876_c0_g1	AT2G27360	GDSL esterase/lipase At2g27360	hydrolase activity	-6.62
TRINITY_DN5904_c0_g1	AT4G00165	Putative lipid-binding protein At4g00165		-6.41
TRINITY_DN44687_c0_g1	ADPG2	Polygalacturonase ADPG2		-6.02
TRINITY_DN6357_c0_g1	PR5K	PR5-like receptor kinase		-5.95
TRINITY_DN9702_c0_g1	JMT	Jasmonate O-methyltransferase		-5.72
TRINITY_DN8238_c0_g2	AT3G03770	Probable inactive leucine-rich repeat receptor-like		-5.70

		protein kinase At3g03770		
TRINITY_DN40496_c0_g1	AT1G04350	1-aminocyclopropane-1-carboxylate oxidase homolog 6		-5.65
**Up-regulated**				
TRINITY_DN209_c0_g2	*ATX5*	Histone-lysine N-methyltransferase ATX5		7.64
TRINITY_DN35748_c1_g1	*LOV1*	Putative inactive disease susceptibility protein LOV1		7.65
TRINITY_DN38698_c0_g1	*BSL1*	Serine/threonine-protein phosphatase BSL1		7.69
TRINITY_DN28687_c0_g1	*GOLS4*	Galactinol synthase 4		7.76
TRINITY_DN40624_c0_g1	*CAT2*	Catalase-2		7.92
TRINITY_DN5170_c0_g1	*AT4G16820*	Phospholipase A1-Ibeta2, chloroplastic		8.16
TRINITY_DN14944_c1_g1	*AT1G78910*	RNA pseudouridine synthase 3, mitochondrial		8.30
TRINITY_DN39320_c0_g1	*NPF7.3*	Protein NRT1/ PTR FAMILY 7.3	transporter activity	9.38
TRINITY_DN6834_c1_g1	*DREB1D*	Dehydration-responsive element-binding protein 1D	DNA binding; transcription regulator activity	9.79
TRINITY_DN137553_c0_g1	*CLPC1*	Chaperone protein ClpC1, chloroplastic	ATP binding; hydrolase activity; nucleic acid binding	12.35
