# Supplementary material for: Ovule Transcriptome Analysis Discloses Deregulation of Genes and Pathways in Sexual and Apomictic Limonium Species (Plumbaginaceae)
Source: Genes (Basel). 2023 Apr 12;14(4):901. doi: 10.3390/genes14040901 (PMC10137852; doi:10.3390/genes14040901)
Supplement: Supplementary file 1 [file genes-14-00901-s001.zip › supplementary table S3.pdf]

Table S3. Uniquely annotated top down- and up-regulated differentially expressed genes (DEGs) in ovules from apomictic *Limonium multiflorum* (M) in stage S1 (1), relative to the control *L. ovalifolium* (o) ovules in either S1 (1) or S2 (2) stages, and *L. multiflorum* (M) in S2 (2) relative to *L. ovalifolium* (o) in S3/S4 (4). DEGs were annotated according to the *Arabidopsis thaliana* reference genome and are presented in ascending order of $\log_2$ fold-change ($\log_2$FC).

Gene ID	Gene Name	Protein Name	Molecular Function	Log_2FC
		**M1o1**		
		**Down-regulated**		
TRINITY_DN5225_c0_g1	*DIR1*	Putative lipid-transfer protein DIR1	lipid binding; protein binding; ion binding	-9.38
TRINITY_DN2097_c0_g1	*CYS5*	Cysteine proteinase inhibitor 5	hydrolase activity	-8.32
TRINITY_DN40200_c0_g1	*AT5G33370*	GDSL esterase/lipase At5g33370	hydrolase activity	-7.96
TRINITY_DN6869_c0_g1	*A6*	Probable glucan endo-1,3-beta-glucosidase A6	hydrolase activity	-7.57
TRINITY_DN9027_c0_g1	*SEP1*	Developmental protein SEPALLATA 1	transcription regulator activity; protein binding; DNA binding	-7.56
TRINITY_DN3934_c0_g1	*AT3G07850*	Exopolygalacturonase clone GBGA483	hydrolase activity	-7.51
TRINITY_DN9597_c0_g2	*MSBP1*	Membrane steroid-binding protein 1	lipid binding	-7.45
TRINITY_DN1467_c0_g1	*GASA7*	Gibberellin-regulated protein 7		-7.43
TRINITY_DN3636_c0_g1	*CER1*	Very-long-chain aldehyde decarbonylase CER1	lyase activity; oxidoreductase activity; ion binding	-7.39
TRINITY_DN7672_c0_g1	*LTP6*	Non-specific lipid-transfer protein 6	lipid binding	-7.20
		**Up-regulated**		
TRINITY_DN31216_c0_g1	*RL6*	Protein RADIALIS-like 6	transcription regulator activity	7.54
TRINITY_DN33030_c0_g1	*GSTU20*	Glutathione S-transferase U20	transferase activity; protein binding; amine binding	7.83
TRINITY_DN41214_c0_g1	*ATM*	Serine/threonine-protein kinase ATM	ATP binding; DNA binding; transferase activity	7.86
TRINITY_DN10435_c0_g1	*ERF5*	Ethylene-responsive transcription factor 5	transcription regulator activity; DNA binding	7.95
TRINITY_DN3975_c0_g1	*IMPA4*	Importin subunit alpha-4	molecular carrier activity; amide binding	8.48

TRINITY_DN11080_c0_g1	*DCD*	Bifunctional D-cysteine desulfhydrase/1-aminocyclopropane-1-carboxylate deaminase, mitochondrial		8.97
TRINITY_DN10398_c0_g3	*NUA*	Nuclear-pore anchor	structural molecule activity	9.25
TRINITY_DN44488_c0_g1	*PRB1*	Pathogenesis-related protein 1	hydrolase activity	10.24
TRINITY_DN68_c0_g1	*PRMT14*	Probable histone-arginine methyltransferase 1.4	transferase activity; protein binding	10.29
TRINITY_DN1353_c0_g1	*ALDH7B4*	Aldehyde dehydrogenase family 7 member B4	oxidoreductase activity	10.89
		**M1o2**		
		**Down-regulated**		
TRINITY_DN40200_c0_g1	*AT5G33370*	GDSL esterase/lipase At5g33370	hydrolase activity	-8.61
TRINITY_DN7779_c0_g1	*AP3*	Floral homeotic protein APETALA 3	transcription regulator activity; protein binding; DNA binding	-8.55
TRINITY_DN185347_c0_g1	*XTH2*	Xyloglucan endotransglucosylase/hydrolase protein 2	hydrolase activity; transferase activity	-8.50
TRINITY_DN5730_c0_g1	*PME38*	Putative pectinesterase/pectinesterase inhibitor 38	hydrolase activity	-8.47
TRINITY_DN10004_c0_g1	*PME67*	Probable pectinesterase 67	hydrolase activity	-8.47
TRINITY_DN8621_c0_g1	*AT59*	Probable pectate lyase 3	ion binding; lyase activity	-8.46
TRINITY_DN19789_c0_g1	*AT3G01270*	Probable pectate lyase 7	ion binding; lyase activity	-8.46
TRINITY_DN253_c1_g1	*COBL10*	COBRA-like protein 10		-8.42
TRINITY_DN6307_c0_g1	*BGAL14*	Beta-galactosidase 14	hydrolase activity; carbohydrate binding	-8.40
TRINITY_DN4212_c0_g1	*NOI4*	Protein NOI4		-8.36
		**Up-regulated**		
TRINITY_DN2100_c1_g3	*CRK8*	Cysteine-rich receptor-like protein kinase 8	ATP binding; transferase activity	5.47
TRINITY_DN11080_c0_g1	*DCD*	Bifunctional D-cysteine desulfhydrase/1-aminocyclopropane-1-carboxylate deaminase, mitochondrial	ion binding; lyase activity; hydrolase activity	5.98
TRINITY_DN6834_c1_g1	*DREB1D*	Dehydration-responsive element-binding protein 1D	transcription regulator activity; DNA binding	6.36

TRINITY_DN1009_c0_g2	*CRK29*	Cysteine-rich receptor-like protein kinase 29	ATP binding; transferase activity	6.50
TRINITY_DN6838_c0_g1	*DRT101*	Phosphoacetylglucosamine mutase	ion binding; isomerase activity	6.62
TRINITY_DN29038_c2_g2	*TPS6*	Alpha,alpha-trehalose-phosphate synthase [UDP-forming] 6	transferase activity; hydrolase activity	6.90
TRINITY_DN27105_c0_g1	*OFUT9*	O-fucosyltransferase 9	transferase activity	6.93
TRINITY_DN44719_c0_g1	*IQM5*	IQ domain-containing protein IQM5		7.44
TRINITY_DN31216_c0_g1	*RL6*	Protein RADIALIS-like 6	transcription regulator activity	7.59
TRINITY_DN3514_c2_g1	*AT1G50180*	Putative disease resistance protein At1g50180	ADP binding; ATP binding	9.77
**M2o4**				
**Down-regulated**				
TRINITY_DN1473_c0_g1	*PRE6*	Transcription factor PRE6	protein binding	-8.52
TRINITY_DN166369_c0_g1	*UGT89A2*	UDP-glycosyltransferase 89A2	transferase activity	-7.67
TRINITY_DN3195_c0_g2	*UBQ4*	Polyubiquitin 4	RNA binding; protein tag; protein binding	-7.65
TRINITY_DN6253_c1_g1	*AT2G32990*	Endoglucanase 11	hydrolase activity	-7.30
TRINITY_DN32294_c0_g1	*A3G2XYLT*	Anthocyanidin 3-O-glucoside 2'''-O-xylosyltransferase	transferase activity	-7.03
TRINITY_DN664_c0_g1	*FACT*	Fatty alcohol:caffeoyl-CoA acyltransferase	transferase activity	-6.78
TRINITY_DN3927_c0_g1	*AT3G28060*	WAT1-related protein At3g28060	transporter activity	-6.67
TRINITY_DN8876_c0_g1	*AT2G27360*	GDSL esterase/lipase At2g27360	hydrolase activity	-6.57
TRINITY_DN25451_c0_g1	*AT2G14610*	Pathogenesis-related protein 1		-6.43
TRINITY_DN11082_c0_g1	*INT4*	Inositol transporter 4	transporter activity	-6.25
**Up-regulated**				
TRINITY_DN344_c1_g1	*AT5G13980*	Probable alpha-mannosidase At5g13980	hydrolase activity; carbohydrate binding; ion binding	13.52
TRINITY_DN175323_c0_g1	*CML32*	Probable calcium-binding protein CML32	ion binding	13.64
TRINITY_DN1517_c0_g1	*DHDPS2*	4-hydroxy-tetrahydrodipicolinate synthase 2, chloroplastic	lyase activity	13.73

TRINITY_DN196_c0_g1	*AT4G18465*	Probable pre-mRNA-splicing factor ATP-dependent RNA helicase DEAH9	ATP binding; hydrolase activity; RNA binding; ATP-dependent activity	13.90
TRINITY_DN271_c0_g1	*LRK10L-1.1*	LEAF RUST 10 DISEASE-RESISTANCE LOCUS RECEPTOR-LIKE PROTEIN KINASE-like 1.1	ATP binding; transferase activity; carbohydrate binding	13.96
TRINITY_DN9987_c1_g1	*PUB7*	U-box domain-containing protein 7	transferase activity	14.13
TRINITY_DN5725_c0_g1	*KCS11*	3-ketoacyl-CoA synthase 11	transferase activity	14.24
TRINITY_DN6909_c0_g1	*UBC29*	Ubiquitin-conjugating enzyme E2 29	ATP binding; transferase activity	14.61
TRINITY_DN9604_c0_g1	*NUDT4*	Nudix hydrolase 4	ion binding; hydrolase activity	15.44
TRINITY_DN5744_c0_g1	*PP2A2*	Protein PHLOEM PROTEIN 2-LIKE A2	ion binding; hydrolase activity	16.87
