# Supplementary material for: Ovule Transcriptome Analysis Discloses Deregulation of Genes and Pathways in Sexual and Apomictic Limonium Species (Plumbaginaceae)
Source: Genes (Basel). 2023 Apr 12;14(4):901. doi: 10.3390/genes14040901 (PMC10137852; doi:10.3390/genes14040901)
Supplement: Supplementary file 1 [file genes-14-00901-s001.zip › supplementary table S4.pdf]

Table S4. Uniquely annotated top down- and up-regulate differentially expressed genes (DEGs) in ovules from apomictic *Limonium multiflorum* in S1 relative to *L. dodartii* in S4 (**M2d4**). DEGs were annotated according to the *Arabidopsis thaliana* reference genome and are presented in ascending order of log_2 fold-change (log_2FC).

Gene ID	Gene Name	Protein Name	Molecular Function	Log_2FC
		**Down-regulated**		
TRINITY_DN194159_c0_g1	*ALDH10A8*	Betaine aldehyde dehydrogenase 1, chloroplastic	oxidoreductase activity	-5.91
TRINITY_DN6424_c0_g1	*HEL*	Hevein-like preproprotein	chitin binding; hydrolase activity	-5.78
TRINITY_DN11809_c0_g1	*HSP70-2*	Heat shock 70 kDa protein 2	ATP-dependent activity; ATP binding; protein binding; protein folding chaperone	-5.55
TRINITY_DN2532_c0_g2	*FOX5*	Berberine bridge enzyme-like 7	FAD binding; oxidoreductase activity	-5.38
TRINITY_DN2544_c0_g1	*KTI5*	Kunitz trypsin inhibitor 5	hydrolase activity	-5.19
TRINITY_DN16670_c0_g2	*RPS4*	Ribosomal protein S4, mitochondrial	RNA binding; structural constituent of ribosome	-5.18
TRINITY_DN9541_c0_g1	*RPS5B*	40S ribosomal protein S5-2	RNA binding; structural constituent of ribosome	-5.13
TRINITY_DN656_c0_g1	*RPS3AB*	40S ribosomal protein S3a-2	RNA binding; structural constituent of ribosome	-5.11
TRINITY_DN3634_c0_g3	*PETA*	Cytochrome f	oxidoreductase activity; heme binding; ion binding; RNA binding	-5.01
TRINITY_DN14609_c0_g1	*EIF4A3*	Eukaryotic initiation factor 4A-III homolog	ATP binding; RNA binding; RNA helicase activity	-4.97
		**Up-regulated**		
TRINITY_DN12965_c2_g1	*CYP81D11*	Cytochrome P450 81D11	heme binding; ion binding; oxidoreductase activity	3.15
TRINITY_DN13513_c0_g1	*DTX45*	Protein DETOXIFICATION 45, chloroplastic	transporter activity	3.30
TRINITY_DN40337_c0_g2	*ARAC2*	Rac-like GTP-binding protein ARAC2	hydrolase activity; GTP binding; protein binding	3.36
TRINITY_DN11327_c0_g1	*FDM3*	Factor of DNA methylation 3		3.41

TRINITY_DN82144_c0_g1	*GLN2*	Glutamine synthetase, chloroplastic/mitochondrial	ATP binding; ligase activity; RNA binding	3.44
TRINITY_DN12987_c0_g1	*PIN5*	Auxin efflux carrier component 5		3.48
TRINITY_DN13321_c0_g2	*CYP71B14*	Cytochrome P450 71B14	heme binding; ion binding; oxidoreductase activity	4.65
TRINITY_DN31216_c0_g1	*RL6*	Protein RADIALIS-like 6	transcription regulator activity	4.83
TRINITY_DN29738_c0_g1	*SMO2-2*	Methylsterol monooxygenase 2-2	ion binding; oxidoreductase activity	4.93
TRINITY_DN13356_c0_g1	*HGO*	Homogentisate 1,2-dioxygenase	oxidoreductase activity; ion binding	8.57
