# Supplementary material for: Ovule Transcriptome Analysis Discloses Deregulation of Genes and Pathways in Sexual and Apomictic Limonium Species (Plumbaginaceae)
Source: Genes (Basel). 2023 Apr 12;14(4):901. doi: 10.3390/genes14040901 (PMC10137852; doi:10.3390/genes14040901)
Supplement: Supplementary file 1 [file genes-14-00901-s001.zip › supplementary table S5.pdf]

Table S5. Uniquely annotated top down- and up-regulate differentially expressed genes (DEGs) in ovules from apomictic *Limonium multiflorum* in stage S2 relative to S1 (**M2m1**). DEGs were annotated according to the *Arabidopsis thaliana* reference genome and are presented in ascending order of log_2 fold-change (Log_2FC).

Gene ID	Gene Name	Protein Name	Molecular Function	Log_2FC
		**Down-regulated**		
TRINITY_DN38876_c0_g1	*AT4G13710*	Probable pectate lyase 15	hydrolase activity; ion binding	-4.61
TRINITY_DN11052_c0_g1	*AT5G37690*	GDSL esterase/lipase At5g37690	hydrolase activity	-4.04
TRINITY_DN25592_c0_g1	*PCO2*	Plant cysteine oxidase 2	oxidoreductase activity; ion binding	-3.43
TRINITY_DN10225_c0_g1	*AT2G23540*	GDSL esterase/lipase At2g23540	hydrolase activity	-3.34
TRINITY_DN4104_c0_g2	*LAC3*	Laccase-3	oxidoreductase activity; ion binding	-3.20
TRINITY_DN4536_c0_g1	*GLP6*	Germin-like protein subfamily 1 member 13	ion binding	-2.81
TRINITY_DN17736_c0_g1	*GASA1*	Gibberellin-regulated protein 1		-2.53
TRINITY_DN5560_c0_g4	*CYP86A1*	Cytochrome P450 86A1	oxidoreductase activity; ion binding; heme binding	-2.45
		**Up-regulated**		
TRINITY_DN157181_c0_g1	*RPL17B*	60S ribosomal protein L17-2	RNA binding; structural constituent of ribosome	7.16
TRINITY_DN156733_c0_g1	*DREB2D*	Dehydration-responsive element-binding protein 2D	transcription regulator activity; DNA binding	7.16
TRINITY_DN39230_c0_g1	*RPL7C*	60S ribosomal protein L7-3	RNA binding; structural constituent of ribosome	7.26
TRINITY_DN28291_c0_g2	*AGL15*	Agamous-like MADS-box protein AGL15	transcription regulator activity; DNA binding; protein binding	7.30
TRINITY_DN43442_c0_g1	*RTNLB8*	Reticulon-like protein B8		7.31
TRINITY_DN2342_c1_g1	*AHA5*	ATPase 5, plasma membrane-type	ATP-dependent activity; ATP binding; transporter activity; ion binding	7.35
TRINITY_DN6665_c0_g1	*AT5G08670*	ATP synthase subunit beta-1, mitochondrial	ATP-dependent activity; ATP binding; transporter activity	7.62

TRINITY_DN4687_c0_g1	*AT5G07830*	Heparanase-like protein 1	hydrolase activity	7.88
TRINITY_DN40215_c0_g1	*CYP18-3*	Peptidyl-prolyl cis-trans isomerase CYP18-3	protein binding; isomerase activity	8.00
TRINITY_DN9971_c0_g1	*MYB80*	Transcription factor MYB80	chromatin binding; transcription regulator activity; DNA binding	8.81
