# Supplementary material for: Ovule Transcriptome Analysis Discloses Deregulation of Genes and Pathways in Sexual and Apomictic Limonium Species (Plumbaginaceae)
Source: Genes (Basel). 2023 Apr 12;14(4):901. doi: 10.3390/genes14040901 (PMC10137852; doi:10.3390/genes14040901)
Supplement: Supplementary file 1 [file genes-14-00901-s001.zip › supplementary table S6.pdf]

Table S6. Uniquely annotated top down- and up-regulated differentially expressed genes (DEGs) in ovules from apomictic *Limonium auriculifolium* (A) in stage S2 (2) relative to S1 (1), and DEGs from ovules in stage S3/S4 (4) relative to either S1 (1) or S2 (2). DEGs were annotated according to the *Arabidopsis thaliana* reference genome and are presented in ascending order of $\log_2$ fold-change (Log_2FC).

Gene ID	Gene Name	Protein Name	Molecular Function	Log_2FC
		**A2a1**		
		**Down-regulated**		
TRINITY_DN8876_c0_g1	*AT2G27360*	GDSL esterase/lipase At2g27360	hydrolase activity	-5.85
TRINITY_DN11184_c0_g1	*AT4G12500*	pEARLI1-like lipid transfer protein 3		-5.46
TRINITY_DN31196_c0_g1	*SBT5.4*	Subtilisin-like protease SBT5.4	hydrolase activity	-5.38
TRINITY_DN604_c0_g1	*AT5G45950*	GDSL esterase/lipase At5g45950	hydrolase activity	-5.25
TRINITY_DN14059_c0_g1	*AT4G28780*	GDSL esterase/lipase At4g28780	hydrolase activity	-5.24
TRINITY_DN2678_c0_g2	*S-ACP-DES1*	Stearoyl-[acyl-carrier-protein] 9-desaturase 1, chloroplastic	oxidoreductase activity; ion binding	-5.00
TRINITY_DN1473_c0_g1	*PRE6*	Transcription factor PRE6	protein binding	-4.87
TRINITY_DN5904_c0_g1	*AT4G00165*	Putative lipid-binding protein At4g00165		-4.63
TRINITY_DN664_c0_g1	*FACT*	Fatty alcohol:caffeoyl-CoA acyltransferase	DNA binding; transcription regulator activity; protein binding; chromatine binding	-4.59
TRINITY_DN166369_c0_g1	*UGT89A2*	UDP-glycosyltransferase 89A2	transferase activity	-4.44
		**Up-regulated**		
TRINITY_DN7650_c0_g1	*VTE5*	Phytol kinase 1, chloroplastic	transferase activity	2.80
TRINITY_DN5906_c1_g1	*AT2G35920*	DExH-box ATP-dependent RNA helicase DExH1	ATP binding; hydrolase activity; RNA binding; helicase activity	2.81
TRINITY_DN41534_c0_g1	*UGT73C7*	UDP-glycosyltransferase 73C7	transferase activity	2.84

TRINITY_DN2871_c0_g1	*AT5G11130*	Probable glycosyltransferase At5g11130	transferase activity	2.87
TRINITY_DN51734_c0_g2	*LTP11*	Non-specific lipid-transfer protein 11	lipid binding	2.89
TRINITY_DN1055_c0_g1	*AT2G04020*	GDSL esterase/lipase At2g04020	hydrolase activity	3.05
TRINITY_DN7962_c0_g1	*PAT1*	Anthranilate phosphoribosyltransferase, chloroplastic	transferase activity	3.08
TRINITY_DN13463_c1_g4	*RBCL*	Ribulose bisphosphate carboxylase large chain	ion binding; oxidoreductase activity; RNA binding; lyase activity	3.30
TRINITY_DN1806_c0_g2	*NDHH*	NAD(P)H-quinone oxidoreductase subunit H, chloroplastic	NAD binding; oxidoreductase activity	3.31
TRINITY_DN5157_c0_g3	*PSBC*	Photosystem II CP43 reaction center protein	chlorophyll binding; oxidoreductase activity; ion binding; RNA binding	3.65
**A4a1**				
**Down-regulated**				
TRINITY_DN28591_c0_g1	*UGT84B1*	UDP-glycosyltransferase 84B1	transferase activity	-4.85
TRINITY_DN7605_c3_g1	*UBQ11*	Polyubiquitin 11	RNA binding; protein tag; protein binding	-4.80
TRINITY_DN184396_c0_g1	*AT1G56220*	Dormancy-associated protein homolog 3		-4.65
TRINITY_DN51734_c0_g2	*LTP11*	Non-specific lipid-transfer protein 11	lipid binding	-4.25
TRINITY_DN166189_c1_g1	*LTP1*	Non-specific lipid-transfer protein 1	protein binding; lipid binding	-4.25
TRINITY_DN105307_c0_g1	*PDF2.3*	Defensin-like protein 1		-4.17
TRINITY_DN4240_c0_g1	*LSU2*	Protein RESPONSE TO LOW SULFUR 2		-4.11
TRINITY_DN45272_c0_g1	*HPD*	4-hydroxyphenylpyruvate dioxygenase	oxidoreductase activity; protein binding; ion binding	-3.95
TRINITY_DN5744_c0_g1	*PP2A2*	Protein PHLOEM PROTEIN 2-LIKE A2		-3.75
TRINITY_DN16864_c1_g1	*EXO*	Protein EXORDIUM		-3.46
**Up-regulated**				

TRINITY_DN10706_c0_g1	*AT2G17570*	Dehydrodolichyl diphosphate synthase 6	transferase activity	3.56
TRINITY_DN31379_c0_g1	*TKPR1*	Tetraketide alpha-pyrone reductase 1	oxidoreductase activity	3.68
TRINITY_DN44687_c0_g1	*ADPG2*	Polygalacturonase ADPG2	hydrolase activity	3.78
TRINITY_DN6156_c0_g1	*PKSA*	Type III polyketide synthase A	transferase activity	3.84
TRINITY_DN6869_c0_g1	*A6*	Probable glucan endo-1,3-beta-glucosidase A6	hydrolase activity	4.03
TRINITY_DN9952_c0_g1	*ABCG26*	ABC transporter G family member 26	ATP binding; transporter activity	4.12
TRINITY_DN22852_c0_g1	*TA1*	Short-chain dehydrogenase reductase ATA1	oxidoreductase activity; protein binding	4.38
TRINITY_DN3406_c2_g2	*XTH22*	Xyloglucan endotransglucosylase/hydrolase protein 22	hydrolase activity; transferase activity	4.39
TRINITY_DN45712_c0_g1	*FAR2*	Fatty acyl-CoA reductase 2, chloroplastic	oxidoreductase activity	4.58
TRINITY_DN6357_c0_g1	*PR5K*	PR5-like receptor kinase	ATP binding; transferase activity	5.18
		**A2a2**		
		**Down-regulated**		
TRINITY_DN51734_c0_g2	*LTP11*	Non-specific lipid-transfer protein 11	lipid binding	-7.16
TRINITY_DN166189_c1_g1	*LTP1*	Non-specific lipid-transfer protein 1	lipid binding; protein binding	-6.96
TRINITY_DN184396_c0_g1	*AT1G56220*	Dormancy-associated protein homolog 3		-6.68
TRINITY_DN5744_c0_g1	*PP2A2*	Protein PHLOEM PROTEIN 2-LIKE A2	ion binding; hydrolase activity	-6.29
TRINITY_DN7605_c3_g1	*UBQ11*	Polyubiquitin 11	RNA binding; protein tag; protein binding	-6.22
TRINITY_DN28591_c0_g1	*UGT84B1*	UDP-glycosyltransferase 84B1	transferase activity	-5.58
TRINITY_DN16864_c1_g1	*EXO*	Protein EXORDIUM		-5.55
TRINITY_DN105307_c0_g1	*PDF2.3*	Defensin-like protein 1		-5.19
TRINITY_DN4240_c0_g1	*LSU2*	Protein RESPONSE TO LOW SULFUR 2		-4.36
TRINITY_DN35822_c0_g1	*PER25*	Peroxidase 25	heme binding; ion binding; oxidoreductase activity	-4.13
		**Up-regulated**		

TRINITY_DN5909_c0_g2	*BG2*	Glucan endo-1,3-beta-glucosidase, acidic isoform	hydrolase activity	5.33
TRINITY_DN5487_c0_g1	*ROPGEF12*	Rop guanine nucleotide exchange factor 12	enzyme regulator activity	5.36
TRINITY_DN8876_c0_g1	*AT2G27360*	GDSL esterase/lipase At2g27360	hydrolase activity	5.41
TRINITY_DN185471_c0_g1	*AT1G30350*	Probable pectate lyase 4	ion binding; lyase activity	5.44
TRINITY_DN11348_c0_g1	*AT4G10955*	GDSL esterase/lipase At4g10955	hydrolase activity	5.48
TRINITY_DN2407_c1_g1	*BZIP34*	Basic leucine zipper 34	DNA binding; transcription regulator activity	5.59
TRINITY_DN12231_c0_g1	*PME13*	Probable pectinesterase/pectinesterase inhibitor 13	hydrolase activity	5.63
TRINITY_DN193961_c0_g1	*CML16*	Probable calcium-binding protein CML16	ion binding; enzyme regulator activity	5.86
TRINITY_DN6869_c0_g1	*A6*	Probable glucan endo-1,3-beta-glucosidase A6	hydrolase activity	6.00
TRINITY_DN6156_c0_g1	*PKSA*	Type III polyketide synthase A	transferase activity	6.20
