# Supplementary material for: Ovule Transcriptome Analysis Discloses Deregulation of Genes and Pathways in Sexual and Apomictic Limonium Species (Plumbaginaceae)
Source: Genes (Basel). 2023 Apr 12;14(4):901. doi: 10.3390/genes14040901 (PMC10137852; doi:10.3390/genes14040901)
Supplement: Supplementary file 1 [file genes-14-00901-s001.zip › supplementary table S7.pdf]

Table S7. Uniquely annotated top down- and up-regulate differentially expressed genes (DEGs) in ovules from apomictic *Limonium ovalifolium* (O) in stage S2 (2) relative to S1 (1) and DEGs from stage S3/S4 (4) relative to either S1 (1) or S2 (2). DEGs were annotated according to the *Arabidopsis thaliana* reference genome and are presented in ascending order of $\log_2$ fold-change (Log_2FC).

Gene ID	Gene Name	Protein Name	Molecular Function	Log_2FC
		**O2o1**		
		**Down**		
TRINITY_DN166189_c1_g1	*LTP1*	Non-specific lipid-transfer protein 1	protein binding; lipid binding	-3,53
TRINITY_DN51734_c0_g2	*LTP11*	Non-specific lipid-transfer protein 11	lipid binding	-3,31
TRINITY_DN16864_c1_g1	*EXO*	Protein EXORDIUM		-3,05
TRINITY_DN2317_c0_g2	*GA2OX6*	Gibberellin 2-beta-dioxygenase 6	metal ion binding; oxidoreductase activity	-2,82
TRINITY_DN5744_c0_g1	*PP2A2*	Protein PHLOEM PROTEIN 2-LIKE A2	ion binding; hydrolase activity	-2,71
TRINITY_DN9463_c0_g1	*EM1*	Em-like protein GEA1		-2,52
TRINITY_DN79538_c1_g1	*COL1*	Zinc finger protein CONSTANS-LIKE 1	transcription regulator activity; metal ion binding; DNA binding; protein binding	-2,41
TRINITY_DN89174_c0_g1	*FQR1*	NAD(P)H dehydrogenase (quinone) FQR1	ribonucleotide binding; oxidoreductase activity	-2,35
TRINITY_DN7605_c3_g1	*UBQ11*	Polyubiquitin 11	RNA binding; protein tag; protein binding	-2,31
TRINITY_DN13979_c0_g2		Probable inactive nicotinamidase At3g16190		-2,3
		**Up**		
TRINITY_DN19440_c0_g1	*PELPK1*	Protein PELPK1		3,99
TRINITY_DN150733_c0_g1	*TBL31*	Protein trichome birefringence-like 31	transferase activity	4,06
TRINITY_DN829_c0_g2	*INVA*	Alkaline/neutral invertase A, mitochondrial	hydrolase activity	4,24
TRINITY_DN8876_c0_g1	*AT2G27360*	GDSL esterase/lipase At2g27360	hydrolase activity	4,27
TRINITY_DN8238_c0_g2	*AT3G03770*	Probable inactive leucine-rich repeat receptor-like protein kinase At3g03770	ATP binding; transferase activity	4,36

TRINITY_DN4747_c0_g1	*EXPB3*	Expansin-B3		4,37
TRINITY_DN14059_c0_g1	*AT4G28780*	GDSL esterase/lipase At4g28780	hydrolase activity	4,38
TRINITY_DN1473_c0_g1	*PRE6*	Transcription factor PRE6	protein binding	4,51
TRINITY_DN604_c0_g1	*AT5G45950*	GDSL esterase/lipase At5g45950	hydrolase activity	5,73
TRINITY_DN5904_c0_g1	*AT4G00165*	Putative lipid-binding protein At4g00165		5,82
**O4o1**				
**Down**				
TRINITY_DN51734_c0_g2	*LTP11*	Non-specific lipid-transfer protein 11	lipid binding	-5,12
TRINITY_DN57_c0_g1	*KIN10*	SNF1-related protein kinase catalytic subunit alpha KIN10	ATP binding; transferase activity; protein binding	-4,95
TRINITY_DN12370_c0_g1	*CSP4*	Cold shock domain-containing protein 4	DNA binding; RNA binding; ion binding	-4,8
TRINITY_DN325_c0_g1	*XI-B*	Myosin-8	ATP-dependent activity; protein binding; ATP binding	-4,13
TRINITY_DN3040_c0_g2	*CIPK3*	CBL-interacting serine/threonine-protein kinase 3	ATP binding; transferase activity	-4,1
TRINITY_DN1497_c0_g1	*RAP2-4*	Ethylene-responsive transcription factor RAP2-4	DNA binding; transcription regulator activity	-3,91
TRINITY_DN8323_c0_g1	*YY1*	Zinc finger transcription factor YY1	transcription regulator activity; metal ion binding; DNA binding	-3,67
TRINITY_DN3733_c0_g2	*IAA18*	Auxin-responsive protein IAA18	transcription regulator activity	-3,63
TRINITY_DN2881_c0_g1	*NHL1*	NDR1/HIN1-like protein 1		-3,59
TRINITY_DN2779_c0_g1	*RPL11A*	60S ribosomal protein L11-1	RNA binding; structural constituent of ribosome	-3,57
**Up**				
TRINITY_DN150733_c0_g1	*TBL31*	Protein trichome birefringence-like 31	transferase activity	5,5
TRINITY_DN12231_c0_g1	*PME13*	Probable pectinesterase/pectinesterase inhibitor 13	hydrolase activity	5,52

TRINITY_DN979_c1_g1	*ATMG00030*	Uncharacterized mitochondrial protein AtMg00030		5,54
TRINITY_DN5904_c0_g1	*AT4G00165*	Putative lipid-binding protein At4g00165		5,79
TRINITY_DN5664_c0_g1	*ATPA*	ATP synthase subunit alpha, chloroplastic	ADP binding; ATP binding; RNA binding; ATP-dependent activity; ion binding	5,9
TRINITY_DN1806_c0_g2	*NDHH*	NAD(P)H-quinone oxidoreductase subunit H, chloroplastic	NAD binding; oxidoreductase activity; quinone binding	5,94
TRINITY_DN1473_c0_g1	*PRE6*	Transcription factor PRE6	protein binding	6,14
TRINITY_DN47860_c0_g2	*PSBE*	Cytochrome b559 subunit alpha	oxidoreductase activity; heme binding; ion binding	6,62
TRINITY_DN604_c0_g1	*AT5G45950*	GDSL esterase/lipase At5g45950	hydrolase activity	6,86
TRINITY_DN2730_c0_g1	*CLPP1*	Chloroplastic ATP-dependent Clp protease proteolytic subunit 1	enzyme binding; ATP-dependent activity; hydrolase activity	7,17
		**O4o2**		
		**Down**		
TRINITY_DN2467_c0_g2	*XTH10*	Probable xyloglucan endotransglucosylase/hydrolase protein 10	hydrolase activity; transferase activity	-4,58
TRINITY_DN57_c0_g1	*KIN10*	SNF1-related protein kinase catalytic subunit alpha KIN10	ATP binding; transferase activity; protein binding	-4,52
TRINITY_DN3040_c0_g2	*CIPK3*	CBL-interacting serine/threonine-protein kinase 3	transferase activity	-4,42
TRINITY_DN12370_c0_g1	*CSP4*	Cold shock domain-containing protein 4	DNA binding; RNA binding; ion binding	-4,28
TRINITY_DN325_c0_g1	*XI-B*	Myosin-8	ATP-dependent activity; protein binding; ATP binding	-4,1
TRINITY_DN21412_c0_g1	*NOP5-1*	Probable nucleolar protein 5-1	RNA binding	-3,87
TRINITY_DN1593_c0_g1	*DSE1*	Protein DECREASED SIZE EXCLUSION LIMIT 1		-3,7

TRINITY_DN6442_c0_g1	*A39*	Aspartic proteinase 39	hydrolase activity	-3,69
TRINITY_DN2779_c0_g1	*RPL11A*	60S ribosomal protein L11-1	RNA binding; structural constituent of ribosome	-3,56
TRINITY_DN1497_c0_g1	*RAP2-4*	Ethylene-responsive transcription factor RAP2-4	transcription regulator activity; DNA binding	-3,46
		**Up**		
TRINITY_DN3634_c0_g1	*NAD7*	NADH dehydrogenase [ubiquinone] iron-sulfur protein 2	oxidoreductase activity; NAD binding	5,18
TRINITY_DN6988_c0_g1	*ND2*	NADH-ubiquinone oxidoreductase chain 2	oxidoreductase activity	5,5
TRINITY_DN979_c1_g1	*ATMG00030*	Uncharacterized mitochondrial protein AtMg00030		5,95
TRINITY_DN3643_c2_g1	*YCF3*	Photosystem I assembly protein Ycf3		6,05
TRINITY_DN5157_c0_g3	*PSBC*	Photosystem II CP43 reaction center protein	chlorophyll binding; oxidoreductase activity; ion binding; RNA binding	6,23
TRINITY_DN13463_c1_g4	*RBCL*	Ribulose bisphosphate carboxylase large chain	ion binding; oxidoreductase activity; RNA binding; lyase activity	6,23
TRINITY_DN1806_c0_g2	*NDHH*	NAD(P)H-quinone oxidoreductase subunit H, chloroplastic	NAD binding; oxidoreductase activity; quinone binding	6,23
TRINITY_DN5664_c0_g1	*ATPA*	ATP synthase subunit alpha, chloroplastic	ADP binding; ATP binding; RNA binding; ATP-dependent activity; transporter activity; ion binding	6,62
TRINITY_DN2730_c0_g1	*CLPP1*	Chloroplastic ATP-dependent Clp protease proteolytic subunit 1	protein binding; ATP-dependent activity; hydrolase activity	6,96
TRINITY_DN47860_c0_g2	*PSBE*	Cytochrome b559 subunit alpha	oxidoreductase activity; heme binding; ion binding	7,14
