# Supplementary material for: Ovule Transcriptome Analysis Discloses Deregulation of Genes and Pathways in Sexual and Apomictic Limonium Species (Plumbaginaceae)
Source: Genes (Basel). 2023 Apr 12;14(4):901. doi: 10.3390/genes14040901 (PMC10137852; doi:10.3390/genes14040901)
Supplement: Supplementary file 1 [file genes-14-00901-s001.zip › supplementary table S8.pdf]

Table S8. Uniquely annotated top down- and up-regulate differentially expressed genes (DEGs) in ovules from sexual *Limonium* plants, namely *L. ovalifolium* (O) relative to the control *L. auriculifolium* (a) in either S1 (1), S2 (2) or S3/S4 (4) stages. DEGs were annotated according to the *Arabidopsis thaliana* reference genome and are presented in ascending order of $\log_2$ fold-change (Log_2FC).

Gene ID	Gene Name	Protein Name	Molecular Function	Log_2FC
		**O1a1**		
		**Down**		
TRINITY_DN5904_c0_g1	*AT4G00165*	Putative lipid-binding protein At4g00165		-6.76
TRINITY_DN604_c0_g1	*AT5G45950*	GDSL esterase/lipase At5g45950	hydrolase activity	-6.22
TRINITY_DN14059_c0_g1	*AT4G28780*	GDSL esterase/lipase At4g28780	hydrolase activity	-5.28
TRINITY_DN1473_c0_g1	*PRE6*	Transcription factor PRE6	protein binding	-5.27
TRINITY_DN8238_c0_g2	*AT3G03770*	Probable inactive leucine-rich repeat receptor-like protein kinase At3g03770	ATP binding; transferase activity	-5.05
TRINITY_DN8876_c0_g1	*AT2G27360*	GDSL esterase/lipase At2g27360	hydrolase activity	-5.05
TRINITY_DN150733_c0_g1	*TBL31*	Protein trichome birefringence-like 31	transferase activity	-4.66
TRINITY_DN4747_c0_g1	*EXPB3*	Expansin-B3		-4.65
TRINITY_DN664_c0_g1	*FACT*	Fatty alcohol:caffeoyl-CoA acyltransferase	transferase activity	-4.57
TRINITY_DN17512_c0_g1	*RRA3*	Arabinosyltransferase RRA3	transferase activity	-4.29
		**Up**		
TRINITY_DN6156_c0_g1	*PKSA*	Type III polyketide synthase A	transferase activity	3.17
TRINITY_DN6357_c0_g1	*PR5K*	PR5-like receptor kinase	ATP binding; transferase activity	3.34
TRINITY_DN4573_c0_g2	*AT5G63180*	Probable pectate lyase 22	metal ion binding; lyase activity	3.65
TRINITY_DN31379_c0_g1	*TKPR1*	Tetraketide alpha-pyrone reductase 1	oxidoreductase activity	3.87
TRINITY_DN6293_c0_g2	*CYP703A2*	Cytochrome P450 703A2	oxidoreductase activity; heme binding; metal ion binding	3.88
TRINITY_DN4485_c0_g1	*CYP704B1*	Cytochrome P450 704B1	oxidoreductase activity; heme binding; metal ion binding	4.24

TRINITY_DN13122_c1_g1	*AT5G03980*	GDSL esterase/lipase At5g03980	hydrolase activity	4.27
TRINITY_DN2985_c0_g1	*GRDP2*	Glycine-rich domain-containing protein 2		4.67
TRINITY_DN9952_c0_g1	*ABCG26*	ABC transporter G family member 26	ATP binding; transporter activity	4.80
TRINITY_DN6869_c0_g1	*A6*	Probable glucan endo-1,3-beta-glucosidase A6	hydrolase activity	4.96
		**O2a2**		
		**Down**		
TRINITY_DN5157_c0_g3	*PSBC*	Photosystem II CP43 reaction center protein	chlorophyll binding; oxidoreductase activity; ion binding; RNA binding	-6.28
TRINITY_DN1806_c0_g2	*NDHH*	NAD(P)H-quinone oxidoreductase subunit H, chloroplastic	NAD binding; oxidoreductase activity	-6.19
TRINITY_DN5664_c0_g1	*ATPA*	ATP synthase subunit alpha, chloroplastic	ADP binding; ATP binding; RNA binding; ATP-dependent activity; ion binding	-6.06
TRINITY_DN47860_c0_g2	*PSBE*	Cytochrome b559 subunit alpha	oxidoreductase activity; heme binding; ion binding	-5.19
TRINITY_DN2730_c0_g1	*CLPP1*	Chloroplastic ATP-dependent Clp protease proteolytic subunit 1	enzyme binding; ATP-dependent activity; hydrolase activity	-5.16
TRINITY_DN13463_c1_g4	*RBCL*	Ribulose bisphosphate carboxylase large chain	ion binding; oxidoreductase activity; RNA binding; lyase activity	-4.61
TRINITY_DN51734_c0_g2	*LTP11*	Non-specific lipid-transfer protein 11	lipid binding	-4.52
TRINITY_DN166189_c1_g1	*LTP1*	Non-specific lipid-transfer protein 1	protein binding; lipid binding	-4.50
TRINITY_DN3643_c2_g1	*YCF3*	Photosystem I assembly protein Ycf3		-4.37
TRINITY_DN10964_c0_g1	*GRXC13*	Glutaredoxin-C13	oxidoreductase activity	-3.87
		**Up**		
TRINITY_DN604_c0_g1	*AT5G45950*	GDSL esterase/lipase At5g45950	hydrolase activity	4.72
TRINITY_DN19440_c0_g1	*PELPK1*	Protein PELPK1		4.77
TRINITY_DN8876_c0_g1	*AT2G27360*	GDSL esterase/lipase At2g27360	hydrolase activity	5.04
TRINITY_DN7672_c0_g1	*LTP6*	Non-specific lipid-transfer protein 6	lipid binding	5.10

TRINITY_DN11184_c0_g1	*AT4G12500*	pEARLI1-like lipid transfer protein 3		5.21
TRINITY_DN2985_c0_g1	*GRDP2*	Glycine-rich domain-containing protein 2		5.36
TRINITY_DN6156_c0_g1	*PKSA*	Type III polyketide synthase A	transferase activity	5.37
TRINITY_DN13122_c1_g1	*AT5G03980*	GDSL esterase/lipase At5g03980	hydrolase activity	5.54
TRINITY_DN5909_c0_g2	*BG2*	Glucan endo-1,3-beta-glucosidase, acidic isoform	hydrolase activity	6.05
TRINITY_DN6869_c0_g1	*A6*	Probable glucan endo-1,3-beta-glucosidase A6	hydrolase activity	7.24
**O4a4**				
**Down**				
TRINITY_DN6442_c0_g1	*A39*	Aspartic proteinase 39	hydrolase activity	-5.23
TRINITY_DN21412_c0_g1	*NOP5-1*	Probable nucleolar protein 5-1	RNA binding	-4.39
TRINITY_DN57_c0_g1	*KIN10*	SNF1-related protein kinase catalytic subunit alpha KIN10	ATP binding; transferase activity; protein binding	-4.15
TRINITY_DN325_c0_g1	*XI-B*	Myosin-8	ATP binding; protein binding; cytoskeletal motor activity	-3.97
TRINITY_DN1893_c2_g1	*OTU4*	OVARIAN TUMOR DOMAIN-containing deubiquitinating enzyme 4	hydrolase activity	-3.81
TRINITY_DN3040_c0_g2	*CIPK3*	CBL-interacting serine/threonine-protein kinase 3	ATP binding; transferase activity	-3.77
TRINITY_DN2343_c4_g1	*IBS1*	Protein IMPAIRED IN BABA-INDUCED STERILITY 1	ATP binding; transferase activity	-3.72
TRINITY_DN74912_c0_g3	*FBL15*	F-box/LRR-repeat protein 15		-3.70
TRINITY_DN4687_c0_g1	*AT5G07830*	Heparanase-like protein 1	hydrolase activity	-3.67
TRINITY_DN12370_c0_g1	*CSP4*	Cold shock domain-containing protein 4	DNA binding; RNA binding; metal ion binding	-3.66
**Up**				
TRINITY_DN30171_c0_g1	*CCMFC*	Cytochrome c biogenesis CcmF C-terminal-like mitochondrial protein		3.92
TRINITY_DN399_c0_g3	*YMF16*	Uncharacterized tatC-like protein ymf16	transporter activity	3.95
TRINITY_DN6988_c0_g1	*ND2*	NADH-ubiquinone oxidoreductase chain 2	oxidoreductase activity	4.06

TRINITY_DN3634_c0_g1	*NAD7*	NADH dehydrogenase [ubiquinone] iron-sulfur protein 2	oxidoreductase activity; quinone binding; NAD binding	4.08
TRINITY_DN1810_c1_g1	*PSBI*	Photosystem II reaction center protein I		4.16
TRINITY_DN1446_c1_g1	*PSAA*	Photosystem I P700 chlorophyll a apoprotein A1	metal cluster binding; metal ion binding; chlorophyll binding; RNA binding; oxidoreductase activity	4.31
TRINITY_DN4450_c0_g2	*CCMB*	Putative cytochrome c biogenesis ccmB-like mitochondrial protein	transporter activity	4.34
TRINITY_DN2890_c1_g1	*RPL14A*	60S ribosomal protein L14-1	RNA binding; structural constituent of ribosome	4.38
TRINITY_DN13463_c1_g4	*RBCL*	Ribulose bisphosphate carboxylase large chain	metal ion binding; oxidoreductase activity; RNA binding; lyase activity	4.71
TRINITY_DN979_c1_g1	*ATMG00030*	Uncharacterized mitochondrial protein AtMg00030		4.71
