# Supplementary material for: Ovule Transcriptome Analysis Discloses Deregulation of Genes and Pathways in Sexual and Apomictic Limonium Species (Plumbaginaceae)
Source: Genes (Basel). 2023 Apr 12;14(4):901. doi: 10.3390/genes14040901 (PMC10137852; doi:10.3390/genes14040901)
Supplement: Supplementary file 1 [file genes-14-00901-s001.zip › supplementary table S9.pdf]

Table S9. Over-representation analysis (ORA) performed by gProfiler of transcription factors (TFs) differentially expressed genes (DEGs) in ovules of *Limonium* plants, namely apomictic *L. multiflorum* (M) and sexual *L. auriculifolium* (a) and *L. ovalifolium* (o). DEGs were filtered by |log2 fold-change (log_2FC)| >2. *Arabidopsis thaliana* most similar homolog of each differentially expressed gene (DEG) was mapped to the respective functional annotation. Significantly (FDR < 0.01) KEGG and WikiPathways metabolic pathways and the respective DEGs from **M1a1**, **M1a2**, **M1o1**, **M1o2**, **M2a4** and **M2o4** (Red: up-regulated DEGs; blue: down-regulated DEGs).

**Gene ID**	**Gene Name**	**Protein Name**	**Log2FC**					
			**M1a1**	**M1a2**	**M1o1**	**M1o2**	**M2a4**	**M2o4**
		**Flower Development [WP:WP618]**						
TRINITY_DN6666_c0_g1	*AG*	Floral homeotic protein AGAMOUS						-2,4
TRINITY_DN9027_c0_g3	*AP1*	Floral homeotic protein APETALA 1	-7,1	-7,2	-7,2	-6,3		
TRINITY_DN7779_c0_g1	*AP3*	Floral homeotic protein APETALA 3					-4,2	-3,2
TRINITY_DN7954_c0_g1	*PI*	Floral homeotic protein PISTILLATA	-8,4	-6,5	-7,1	-8,3		-3,4
TRINITY_DN12350_c0_g1	*RAP2-7*	Ethylene-responsive transcription factor RAP2-7					-4,2	
TRINITY_DN9027_c0_g1	*SEP1*	Developmental protein SEPALLATA 1	-7,9		-7,6	-8,1		
TRINITY_DN31040_c0_g1	*SEP3*	Developmental protein SEPALLATA 3					-2,4	
TRINITY_DN2251_c0_g1	*SOC1*	MADS-box protein SOC1						
TRINITY_DN29363_c0_g1	*SVP*	MADS-box protein SVP						
		**Flower Development (Initiation) [WP:WP2108]**						
TRINITY_DN6666_c0_g1	*AG*	Floral homeotic protein AGAMOUS						-2,4
TRINITY_DN9027_c0_g3	*AP1*	Floral homeotic protein APETALA 1	-7,1	-7,2	-7,2	-6,3		
TRINITY_DN2754_c0_g1	*AP2*	Floral homeotic protein APETALA 2						2,1
TRINITY_DN7779_c0_g1	*AP3*	Floral homeotic protein APETALA 3					-2,5	-3,2
TRINITY_DN7954_c0_g1	*PI*	Floral homeotic protein PISTILLATA						-3,4
TRINITY_DN12350_c0_g1	*RAP2-7*	Ethylene-responsive transcription factor RAP2-7	6,4		6,5	4,6	4,6	
TRINITY_DN31040_c0_g1	*SEP3*	Developmental protein SEPALLATA 3					-2,4	

**Lysine degradation [KEGG:00310]**						
TRINITY_DN8133_c0_g1	*ASHR1*	Histone-lysine N-methyltransferase ASHR1				10,1
TRINITY_DN1656_c0_g1	*ATXR3*	Histone-lysine N-methyltransferase ATXR3				2,1
TRINITY_DN15991_c0_g1	*ATXR5*	Histone-lysine N-methyltransferase EZA1				6,8
TRINITY_DN14929_c0_g1	*EZA1*	Histone-lysine N-methyltransferase EZA1				2,9
TRINITY_DN22984_c0_g1	*SUVH6*	Histone-lysine N-methyltransferase, H3 lysine-9 specific SUVH6				7,2
TRINITY_DN393_c0_g2	*SUVH9*	Histone-lysine N-methyltransferase family member SUVH9				10,4
TRINITY_DN10785_c0_g1	*SUVR3*	Histone-lysine N-methyltransferase SUVR3				7,1
**MAPK signaling pathway - plant [KEGG:04016]**						
TRINITY_DN11495_c0_g1	*EIN4*	Protein EIN4				10,5
TRINITY_DN9787_c0_g1	*AT1G66345*	Pentatricopeptide repeat-containing protein At1g66345				9,6
TRINITY_DN7020_c3_g1	*MYC2*	Transcription factor MYC2				2,6
TRINITY_DN10939_c0_g2	*WRKY29*	Probable WRKY transcription factor 29				8,1
TRINITY_DN42768_c0_g1	*WRKY33*	Probable WRKY transcription factor 33				2,8
**Plant hormone signal transduction [KEGG:04075]**						
TRINITY_DN5251_c0_g1	*AHK3*	Histidine kinase 3	2,6	2,4	2,6	9,8
TRINITY_DN5309_c0_g1	*AHK4*	Histidine kinase 4		-3,8	-2,2	
TRINITY_DN106_c0_g1	*ARF1*	Auxin response factor 1				2,7
TRINITY_DN21549_c0_g1	*ARR11*	Two-component response regulator ARR11				8,3
TRINITY_DN12286_c0_g1	*ARR17*	Two-component response regulator ARR17			-4,1	
TRINITY_DN7878_c1_g2	*ARR9*	Two-component response regulator ARR9	-2,3	-2,4		8,6
TRINITY_DN13746_c0_g1	*BZR2*	Protein BRASSINAZOLE-RESISTANT 2				7,6
TRINITY_DN56546_c0_g1	*DPBF3*	ABSCISIC ACID-INSENSITIVE 5-like protein 2				6,7
TRINITY_DN11495_c0_g1	*EIN4*	Protein EIN4				10,5

TRINITY_DN5700_c2_g1	*ERF2*	Ethylene-responsive transcription factor 2		2,2	10,8
TRINITY_DN9787_c0_g1	*AT1G66345*	Pentatricopeptide repeat-containing protein At1g66345			9,6
TRINITY_DN5071_c0_g1	*GBF4*	G-box-binding factor 4			11,7
TRINITY_DN6334_c0_g1	*IAA14*	Auxin-responsive protein IAA14	-3,4	-2,8	
TRINITY_DN16729_c0_g1	*IAA27*	Auxin-responsive protein IAA27		-4,2	-3,1
TRINITY_DN243_c0_g4	*IAA3*	Auxin-responsive protein IAA3		-2,2	
TRINITY_DN11282_c0_g2	*IAA7*	Auxin-responsive protein IAA7		-7,1	-5
TRINITY_DN7020_c3_g1	*MYC2*	Transcription factor MYC2		2,8	2,6
TRINITY_DN9310_c0_g1	*NPR5*	Regulatory protein NPR5		-2	
TRINITY_DN2328_c0_g1	*TGA1*	Transcription factor TGA1			11,1
TRINITY_DN10239_c0_g1	*TGA2*	Transcription factor TGA2	2,1		2,8
TRINITY_DN20363_c0_g1	*TGA6*	Transcription factor TGA6			3,1
