# Supplementary material for: Ovule Transcriptome Analysis Discloses Deregulation of Genes and Pathways in Sexual and Apomictic Limonium Species (Plumbaginaceae)
Source: Genes (Basel). 2023 Apr 12;14(4):901. doi: 10.3390/genes14040901 (PMC10137852; doi:10.3390/genes14040901)
Supplement: Supplementary file 1 [file genes-14-00901-s001.zip › Table_S1.pdf]

Table S1. Quantification of basic quality and completeness metrics of *Limonium de novo* transcriptome assembly. Samples of apomictic (*L. multiflorum*), facultative apomictic (*L. dodartii*), sexual (*L. ovalifolium*, *L. auricolifolium*) in S1, S2, S1/S2, S3/S4 and S4 stages were combined to perform a *de novo* transcriptome assembly using Trinity software's.

**Basic Metrics**	**Values**
Total trinity unigenes	162520
Total trinity transcripts (isoforms)	315983
Percent GC (%)	42.94
Contig N10	4592
Contig N20	3604
Contig N30	2998
Contig N40	2528
Contig N50	2128
**Completeness**	
Total number of core genes queried	2326
Number of core genes detected	
Complete	2090 (89.85%)
Complete + Partial	2159 (92.82%)
Number of missing core genes	167 (7.18%)
Average number of orthologs per core gene	2.93
% of detected core genes that have more than 1 ortholog	70.81
Scores in BUSCO format	C:89.8% [S:26.2%, D:63.6%], F:3.0%, M:7.2%
