# Supplementary figures and images for: Ovule Transcriptome Analysis Discloses Deregulation of Genes and Pathways in Sexual and Apomictic Limonium Species (Plumbaginaceae)
Source: Genes (Basel). 2023 Apr 12;14(4):901. doi: 10.3390/genes14040901 (PMC10137852; doi:10.3390/genes14040901)

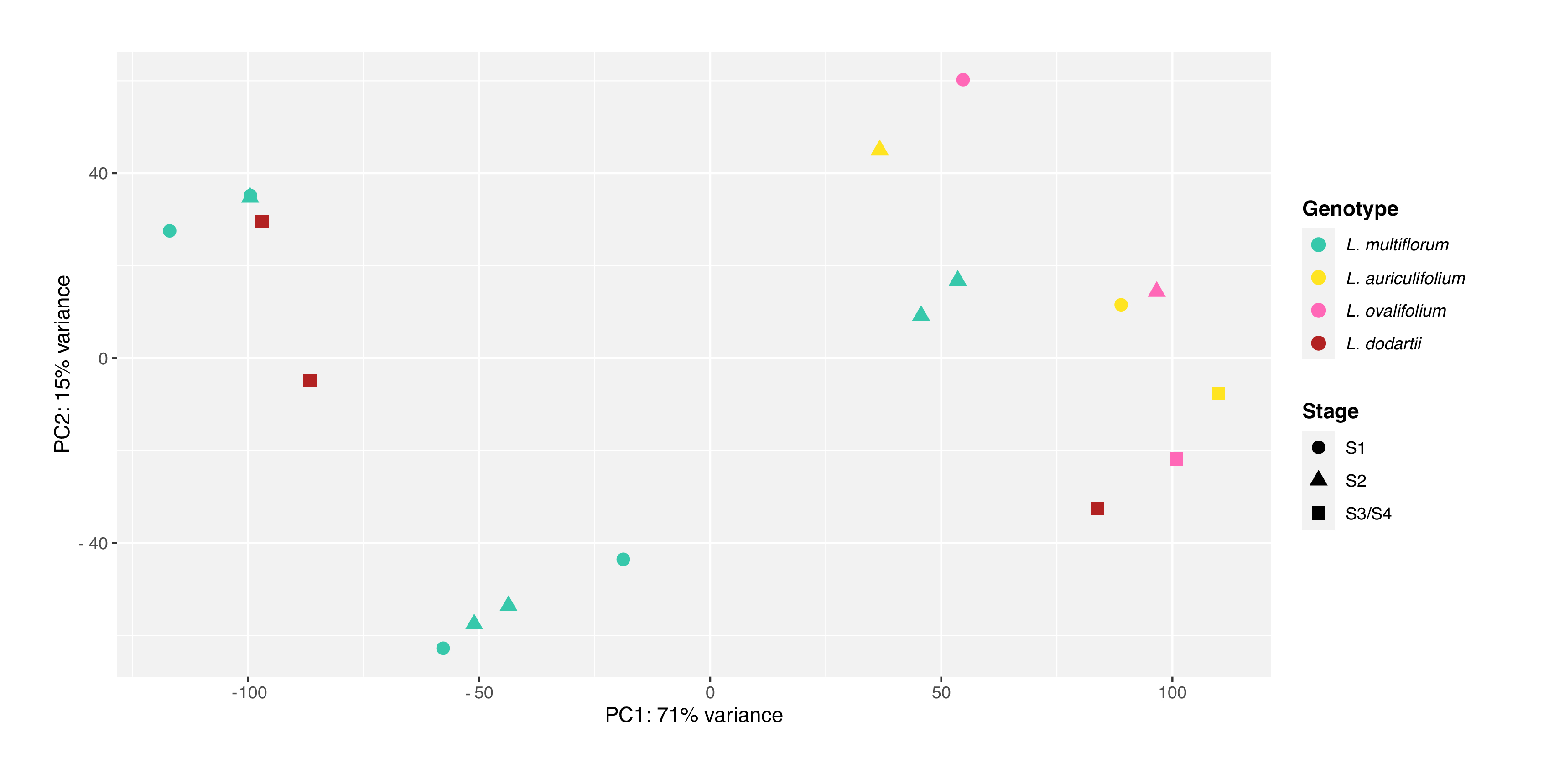

Supplement: Supplementary file 1 [file genes-14-00901-s001.zip › supplementary figure S1.jpeg]

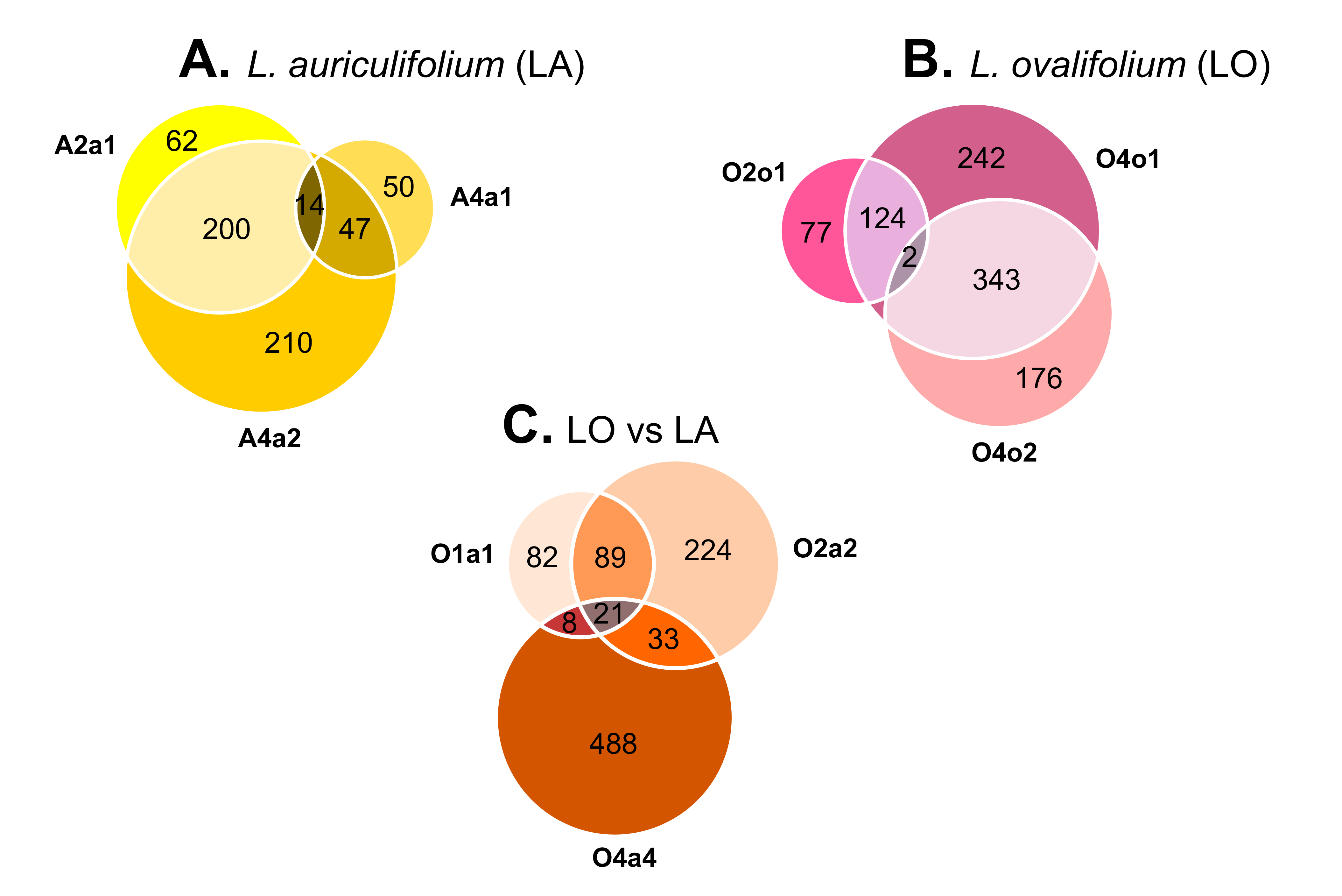

Supplement: Supplementary file 1 [file genes-14-00901-s001.zip › supplementary figure S2.jpeg]

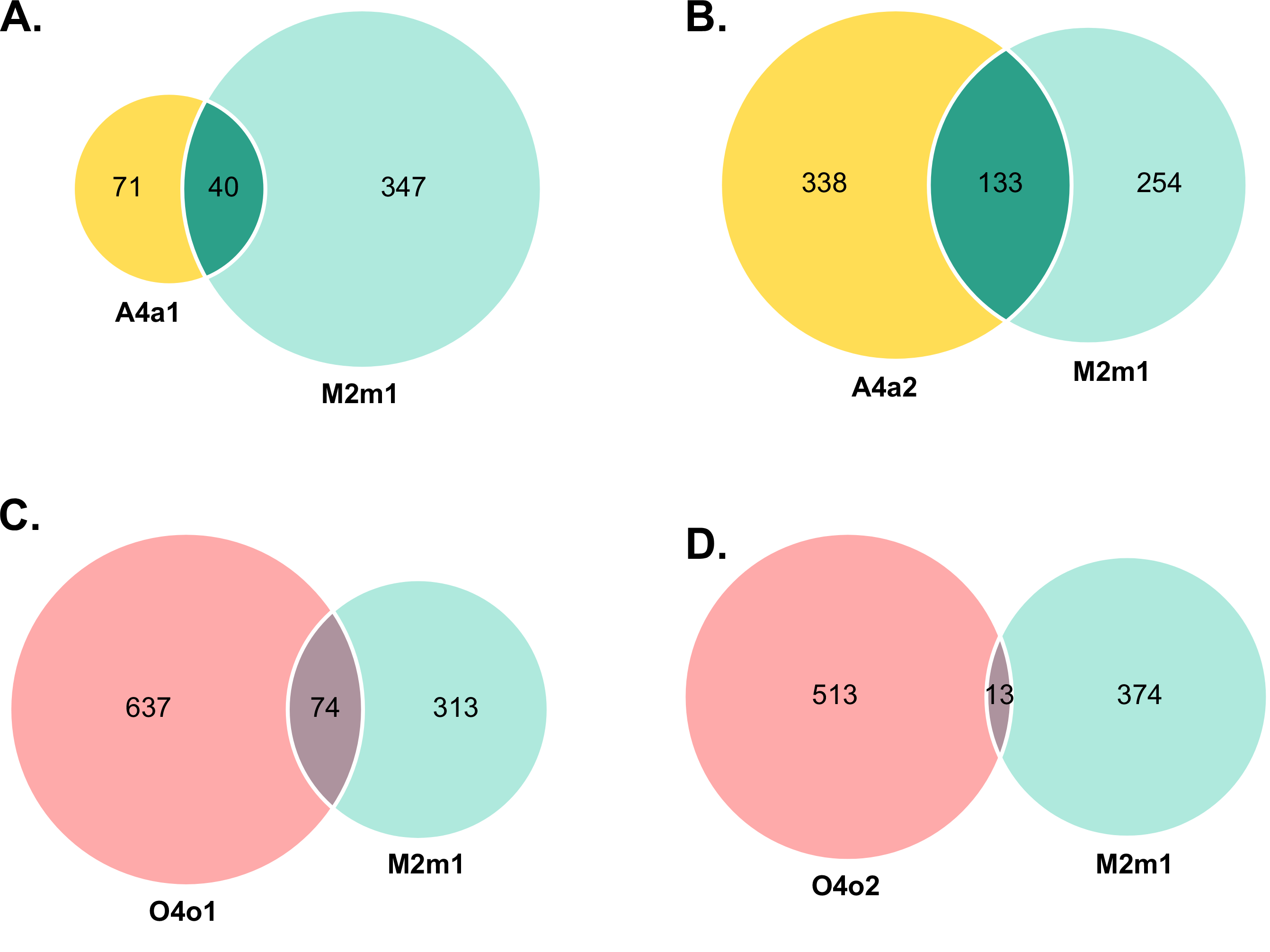

Supplement: Supplementary file 1 [file genes-14-00901-s001.zip › supplementary figure S3.jpeg]

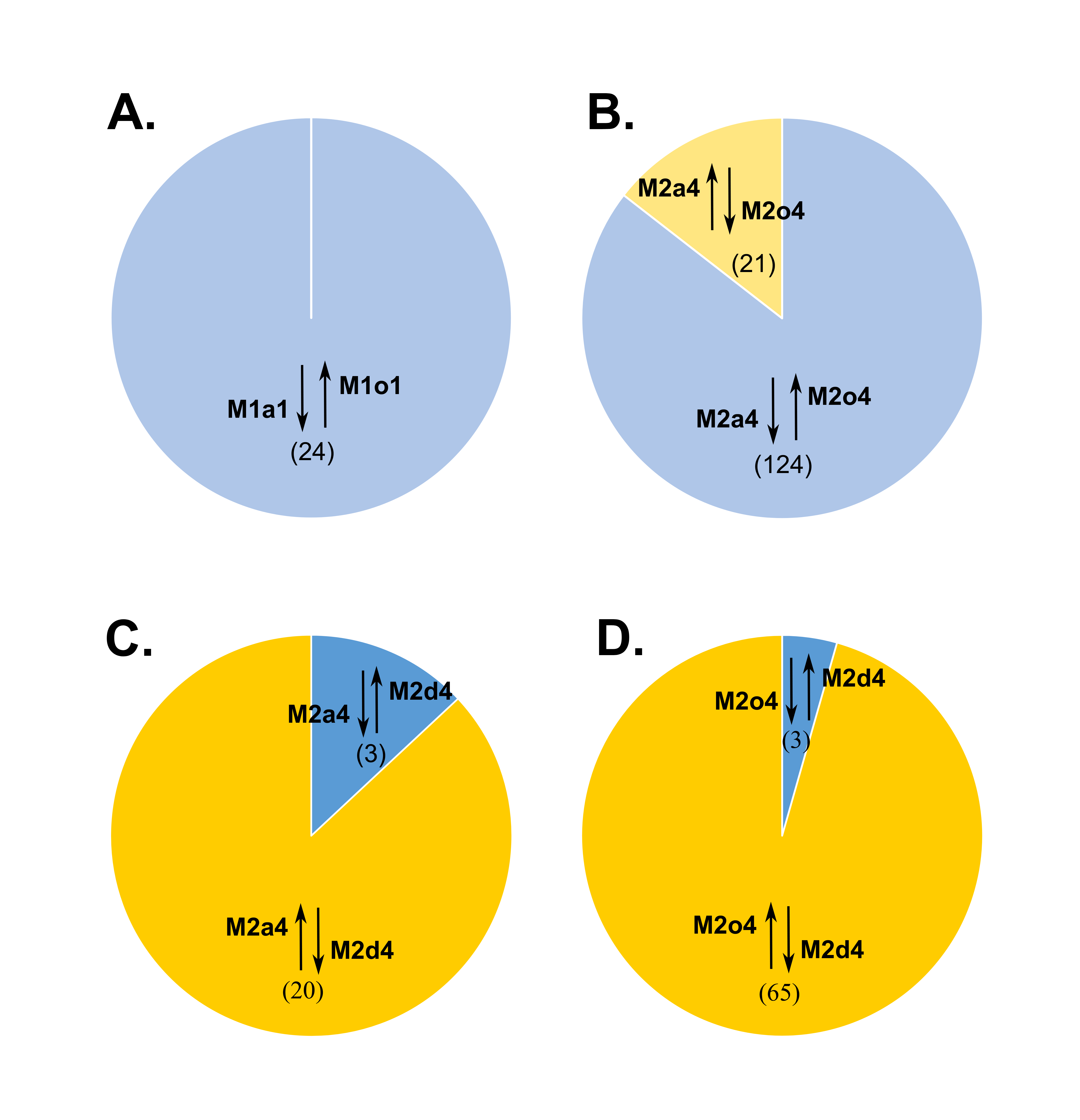

Supplement: Supplementary file 1 [file genes-14-00901-s001.zip › supplementary figure S4.jpeg]

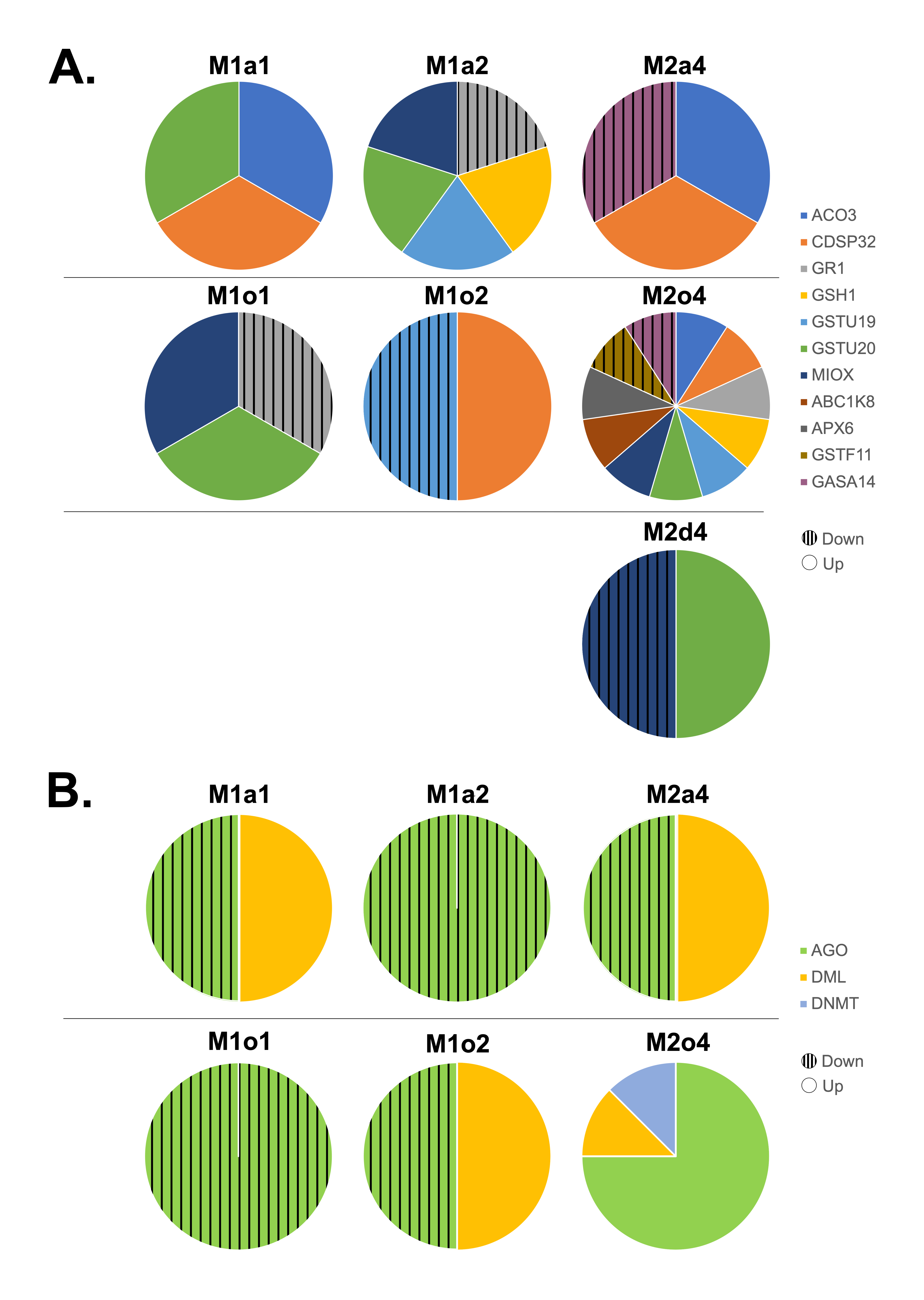

Supplement: Supplementary file 1 [file genes-14-00901-s001.zip › supplementary figure S5.jpeg]
